# A new mRNA structure prediction based approach to identifying improved signal peptides for bone morphogenetic protein 2

**DOI:** 10.1186/s12896-024-00858-1

**Published:** 2024-05-23

**Authors:** Piers Wilkinson, Brian Jackson, Hazel Fermor, Robert Davies

**Affiliations:** 1 Department of Mechanical Engineering, Institute of Medical and Biological Engineering, University of Leeds, Leeds, UK; 2 Faculty of Biological Sciences, University of Leeds, Leeds, UK; 3 School of Biomedical Sciences, Faculty of Biological Sciences, University of Leeds, Leeds, UK; 4 Oral Biology, Faculty of Medicine and Health, University of Leeds, Leeds, UK

**Keywords:** Signal peptides_1_, Bone morphogenetic protein_2_, RNA structure_3_, Protein engineering_4_, Synthetic biology_5_, Gene therapy_6_, Regenerative medicine_7_

## Abstract

**Background:**

Signal peptide (SP) engineering has proven able to improve production of many proteins yet is a laborious process that still relies on trial and error. mRNA structure around the translational start site is important in translation initiation and has rarely been considered in this context, with recent improvements in *in silico* mRNA structure potentially rendering it a useful predictive tool for SP selection. Here we attempt to create a method to systematically screen candidate signal peptide sequences *in silico* based on both their nucleotide and amino acid sequences. Several recently released computational tools were used to predict signal peptide activity (SignalP), localization target (DeepLoc) and predicted mRNA structure (MXFold2). The method was tested with Bone Morphogenetic Protein 2 (BMP2), an osteogenic growth factor used clinically for bone regeneration. It was hoped more effective BMP2 SPs could improve BMP2-based gene therapies and reduce the cost of recombinant BMP2 production.

**Results:**

Amino acid sequence analysis indicated 2,611 SPs from the TGF-β superfamily were predicted to function when attached to BMP2. mRNA structure prediction indicated structures at the translational start site were likely highly variable. The five sequences with the most accessible translational start sites, a codon optimized BMP2 SP variant and the well-established hIL2 SP sequence were taken forward to in vitro testing. The top five candidates showed non-significant improvements in BMP2 secretion in HEK293T cells. All showed reductions in secretion versus the native sequence in C2C12 cells, with several showing large and significant decreases. None of the tested sequences were able to increase alkaline phosphatase activity above background in C2C12s. The codon optimized control sequence and hIL2 SP showed reasonable activity in HEK293T but very poor activity in C2C12.

**Conclusions:**

These results support the use of peptide sequence based *in silico* tools for basic predictions around signal peptide activity in a synthetic biology context. However, mRNA structure prediction requires improvement before it can produce reliable predictions for this application. The poor activity of the codon optimized BMP2 SP variant in C2C12 emphasizes the importance of codon choice, mRNA structure, and cellular context for SP activity.

## Background

 Signal peptides (SPs), also known as signal sequences, are the region of secreted proteins that target them to a secretion pathway. In eukaryotes most secreted proteins use the signal recognition particle (SRP) secretion pathway, relying on short N-terminal signal peptides that induce co-translational translocation into the endoplasmic reticulum. The SRP pathway and its SPs have seen a great deal of basic research over several decades and much is understood of their function (reviewed elsewhere [1]), however SP sequences are highly diverse and it is not understood why some function better than others in specific contexts.

A rarely considered aspect of SP behaviour is the influence of its nucleotide sequence on translation initiation. The SP nucleotide sequence lies at the 5’ end of coding sequence and contains the start codon and the last nucleotide of the Kozak sequence. Crucially, it is likely to form secondary structures with the latter segment of the 5’ UTR, the region containing the ribosomal attachment site. mRNA structure around the ribosomal attachment site is now established as an important mediator of translation initiation, with stable secondary structure in the region known to inhibit the process [2–6]. It therefore seems prudent to consider mRNA structure alongside amino acid sequence when selecting SP candidates. However, the large majority of SP optimization or substitution approaches do not do so, likely due to difficulties in predicting structure *in silico* or establishing it empirically. There have been a handful of attempts to integrate these factors in the past using *in silico* methods, though these were held back by difficulties in predicting mRNA structure and saw little success [7, 8]. The years since these attempts have seen rapid improvement in computational techniques due to advances in machine learning [9–13]. It was hoped the new generation of tools using these methods might offer improved performance for both amino acid sequence based SP prediction and mRNA structure prediction.

The method was tested with Bone Morphogenetic Protein 2 (BMP2), an osteogenic growth factor in the TGF-β family that plays important roles in bone development, homeostasis and healing [14–19]. BMP2 has seen extensive use in regenerative approaches for bone healing and other orthopaedic applications, both as a recombinant protein and a gene therapy [20, 21]. The aim was to ameliorate methodologies to improve BMP2 secretion, hopefully improving the effectiveness of BMP2 gene therapies and reduce the cost of recombinant BMP2 production. Only one prior publication has attempted to identify improved SPs for BMP2 using a non-systematic approach, failing to identify any SPs offering improved secretion [22].

Here we present a new method to identify SPs to improve BMP2 secretion. The method employs various up-to-date computational tools from other authors to predict SP activity based on amino acid and nucleotide sequences, with particularly attention paid to the predicted mRNA structure at the ribosomal attachment site. A subset of the top results and two manually selected sequences of interest were then tested in vitro in two cells lines, with protein secretion and osteogenic activity investigated. We report the strengths and weaknesses of the technique and suggest several approaches to improve the method.

## Methods

### Acquisition of signal peptide amino acid sequences and creation of the *in silico *fusion peptide library

The protein sequences for all identified members of the TGF-β superfamily in all species were downloaded from the UniProt database as an XML file on 13/10/2022 (URL: https://www.uniprot.org/uniprotkb/?query=family:%22TGF-beta+family%22&sort=score) [23] . The SP amino acid sequences of proteins annotated with SPs were isolated and attached to the hBMP2 propeptide amino acid sequence, creating an *in silico* library of fusion proteins. Nucleotide sequences corresponding to the SP amino acid sequences were retrieved by isolating source database information and accession numbers from the UniProt data. The various nucleotide sequence databases cited (NCBI nucleotide, EBI-ENA, Ensembl, WormBase ParaSite [24–27]) were then accessed by their application programming interfaces to retrieve the relevant sequence data. After retrieval, the nucleotide sequence dataset was subjected to an availability and validity check using the following criteria: nucleotide sequence successfully retrieved, sequence in frame, sequence begins with a start codon, and translated nucleotide sequence matches the UniProt protein sequence.

### Prediction of signal peptide function and localisation with signalP and deepLoc

The *in silico* fusion proteins were analysed with signal peptide prediction software SignalP 6.0 (Technical University of Denmark) to predict if they would be recognised by secretion machinery [11]. The fast model was chosen due to substantially reduced completion time for the large data set used here. SignalP gives a score for the predicted likelihood of the sequence containing a functional eukaryotic SRP pathway signal peptide (called the Sec/SPI score) between 0 and 1, with sequences given higher scores considered more likely to contain an SRP signal peptide. An exclusion threshold of Sec/SPI < 0.5 was set.

The signal peptide targeting predictor DeepLoc 2.0 (Technical University of Denmark) was used to predict the localisation targets of the SPs [12]. DeepLoc provides predictions of localisation to various intracellular targets and the extracellular space, giving a score of 0 to 1 for each location with higher values indicating increased confidence in the prediction. An exclusion threshold of a < 0.5 for extracellular localisation was set.

### Manually selected sequences

The expression optimisation tool “Translation Initiation coding region designer” (TISIGNER, https://tisigner.com/tisigner) was used to design a codon optimised version of the endogenous hBMP2 SP. TISIGNER prioritises minimal mRNA 5’ opening energy rather than using a more standard tRNA availability based approach (see Bhandari et al. for a full description of the method [28]). Host organism was specified as “Other” and the following promoter and 5’ UTR sequence from the intended pVax plasmid vector was entered to allow mRNA structure prediction (5’ – 3’ orientation):

GTGATGCGGTTTTGGCAGTACATCAATGGGCGTGGATAGCGGTTTGACTCACGGGGATTTCCAAGTCTCCACCCCATTGACGTCAATGGGAGTTTGTTTTGGCACCAAAATCAACGGGACTTTCCAAAATGTCGTAACAACTCCGCCCCATTGACGCAAATGGGCGGTAGGCGTGTACGGTGGGAGGTCTATATAAGCAGAGCTCTCTGGCTAACTAGAGAACCCACTGCTTACTGGCTTATCGAAATTAATACGACTCACTATAGGGAGACCCAAGCTGGCTAGCGTTTAAACTTAAGCTTGGTACCGAGCTCGGATCCACTAGTCCAGTGTGGTGGAATTCGGCTTGCCACC.

The full hBMP2 mature transcript nucleotide sequence from the NCBI nucleotide database (Accession: NM_001200.4) was then entered for optimisation.

The signal peptide nucleotide sequence from the hIL2 mRNA was taken from the hIL2 references sequence on NCBI nucleotide (Accession: NM_000586.4).

### Creation of *in silico *predicted mRNAs and structure, stability and opening energy prediction

To predict the structure and stability of the fusion proteins’ mRNAs when expressed from the intended pVax_BMP2 based plasmid vector (sequence available at https://www.ncbi.nlm.nih.gov/nuccore/MK433563.1), *in silico* predicted mRNAs were created. In 5’ to 3’ orientation these contained the 5’ UTR of pVax_BMP2, the SP of interest, the hBMP2 propeptide, the pVax_BMP2 3’ UTR, the BGH poly-A sequence to 20 bp downstream of the AATAA polyadenylation site, then a 150 bp poly A tail. The resulting predicted mRNA sequences were ~ 1.7 kb in length.

The RNA secondary structure predictor MXFold2 (Department of Biosciences and Informatics, Keio University, Japan) was used to predict the secondary structures and calculate the stability of the predicted mRNAs [13]. Stability was quantified with minimum free energy (MFE) values, which account for the predicted energetic contribution of every feature in the predicted secondary structure [13]. To calculate opening energy the MXFold2-generated secondary structures were submitted to RNAeval (see ViennaRNA package [29]) to provide the predicted energetic contribution of each bond in the MXFold2 generated structure. Opening energy was predicted by extracting the energy values for the ± 15nt segment surrounding the start codon (previously established as a relevant window by others [3–5]) using a python script. A simple metric was devised to simultaneously consider the predicted opening energy and total mRNA stability values, generating a metric termed SP score.

$$SP score=\frac{MFE}{opening\ energy}$$

Higher SP scores indicate larger predicted MFE and smaller predicted opening energy; the hypothetically favourable combination of properties for maximising expression.

### Additional *in silico *analysis

RNA structure visualisation was performed with the Forna web app. (http://rna.tbi.univie.ac.at/forna/). See Kerpedjiev et al. for details [30]. Nucleotide and amino acid sequence alignments were performed in SnapGene (SnapGene software, www.snapgene.com). The final 7 candidates and the endogenous hBMP2 SP were submitted to the SignalP 6 web app (https://services.healthtech.dtu.dk/services/SignalP-6.0/) to provide predictions of region boundaries [11]. “Organism: Eukarya”, “Output format: Long” and “Model mode: Slow” were specified prior to running the model.

### Plasmids

The plasmids pVax_BMP2 and pVax_BMP7 were kindly provided by Dr Georg Feichtinger (see Feichtinger et al. for details [31]). The pVax_GFP control plasmid was produced from pVax_BMP2 by restriction cloning. Briefly, the BMP2 CDS was removed and replaced with EGFP excised from the pCAG_GFP plasmid previously purchased from Addgene (https://www.addgene.org/11150/). Novel SP fragment synthesis, restriction cloning into pVax_BMP2 and endotoxin free maxi-prep scale up were outsourced using the Genewiz TurboGENE 7 Day service (Azenta life sciences).

### Cell culture

HEK293T cells were kindly provided by Dr Brian Jackson. The cells were maintained in DMEM high glucose (D6429, Sigma), 10% FBS (EU-000-F, Seralab), 2 mM L-Glutamine (G7513, Sigma), 1% v/v penicillin/streptomycin. C2C12 cells were purchased from ATCC (CRL-1772). The cells were maintained in DMEM high glucose with 4mM L-Glutamine and 5% FBS, without antibiotics. Both lines were maintained at 37^0^C in a 5% CO2 atmosphere and passaged twice a week during maintenance periods.

HEK293T cells were seeded in 24-well plates at 3 × 10^5^ cells/well in 1 ml of complete DMEM, 24 h prior to transfection. Cells were transfected using Lipofectamine 3000 (L3000001, Thermo Fisher Scientific) at the manufacturer recommended high concentration. After 24 h an additional 1 ml of complete DMEM was added to each well to ensure the media was not exhausted before harvesting. Media was harvested 48 h after transfection. SP panel experiments contained 9 groups with each transfected with one of the following plasmids: the top 5 results from the in silico screen, the two manually selected SP sequences (SP hBMP2 TISIGNER and SP hIL2), a pVax_BMP2 positive control and a pVax_GFP negative control. Transfections were always performed in technical duplicate with two wells per group.

C2C12 cells were seeded in 24 well plates at 6 × 10^4^ cells/well in 1 ml of complete C2C12 media. The cells were transfected after 24 h as described above. Experimental groups were the same as those in the HEK293T experiment with the addition of a co-transfection pVax_BMP2 + pVax_BMP7 osteogenesis positive control group (effectiveness previously established by others [31]). In this group 250ng of each plasmid was used per well to match the 500ng used in the other groups. 24 h after lipofection the cells were media changed with 1 ml of complete media supplemented with 10 µg/ml heparin (H3149, Sigma Aldrich) to reduce interaction with heparan sulphate proteoglycans that would otherwise rapidly clear hBMP2 from solution [32, 33]. After a further 24 h (48 h after lipofection) the media was harvested for BMP2 ELISA and new heparin-supplemented media added. The plates were maintained in heparin-supplemented media until 7 days post transfection, with media changes every 2/3 days. On day 7 the wells were washed with PBS and the plates stored at -80^0^C for up to 48 h prior to quantitative alkaline phosphatase (ALP) assay. Transfections were always performed in technical duplicate, with two wells per condition. Each well was considered a separate sample for ELISA and ALP assays.

### hBMP2 ELISA

hBMP2 capture and biotin-conjugated detection antibodies, CHO-derived rhBMP2 standard and streptavidin-HRP working solution from the hBMP2 DuoSet ELISA system (DY355, R&D systems) were used for all experiments. Nunc MaxiSorp ELISA plates (M9410, Merck), 10% BSA ELISA reagent diluent/blocking solution concentrate (DY995, R&D systems) and TMB substrate (421,501, Biolegend) were purchased separately and used for all experiments. ELISAs were performed according to the hBMP2 DuoSet ELISA system manufacturer recommendations. rhBMP2 standard curves were made up in the appropriate complete media. A Wellwash microplate washer (5,165,000, Thermo Fisher Scientific) was used for all washes. ELISAs were always performed in technical duplicate (two wells per sample). The mean value from the two wells was used for data analysis.

### Quantitative ALP assay

Nitrophenol phosphate (NPP) quantitative ALP assays were performed to assess osteogenesis in the C2C12s seven days after transfection. Cells were washed with PBS then lysed with 100ul lysis solution (0.5% Triton X-100 in deionized water) per well with 250RPM radial shaking for 1 h. 10ul from each well was transferred to a new 96 well plate, then a 90ul of NPP working solution (5mM NPP (4876, EMD Millipore), 0.5 M 2-AMP (A9199, Sigma Aldrich), 2mM MgCl2 (25108.260, VWR chemical), pH 10.3 in deionised water) was added to each well. A 4-Nitrophenol (4NP) end-product standard curve of 10–200µM (4NP to concentration(1048, Sigma Aldrich), 0.5 M 2-AMP, 2mM MgCl_2_, pH 10.3 buffer) was run simultaneously to allow calculation of the final 4NP concentration in each well. Plates were incubated at room temperature for 45 min then absorbances at 405 nm and a 600 nm wavelength control were measured using a microplate spectrophotometer (Multiskan GO microplate reader, Thermo Fisher). ALP assays were performed in technical triplicate (three wells per sample). Mean values were used for analysis.

### Statistics

ELISA and ALP data were normalised to the positive control group prior to analysis. For ELISAs the positive control was the pVax_BMP2 group, while for ALP assays the positive control was the pVax_BMP2 + pVax_BMP7 group. For HEK293T ELISA data *N* = 7, for the C2C12 ELISA and ALP data *N* = 3. The normalised data were then tested for normality using Shapiro-Wilk tests. All data were found to be normally distributed, and significance was tested using one-way ANOVAs with selected Dunnett’s multiple comparison tests between the positive control and other groups to improve power. All statistical tests were performed in GraphPad Prism 9.3.1 (GraphPad Software).

**Fig. 1:**
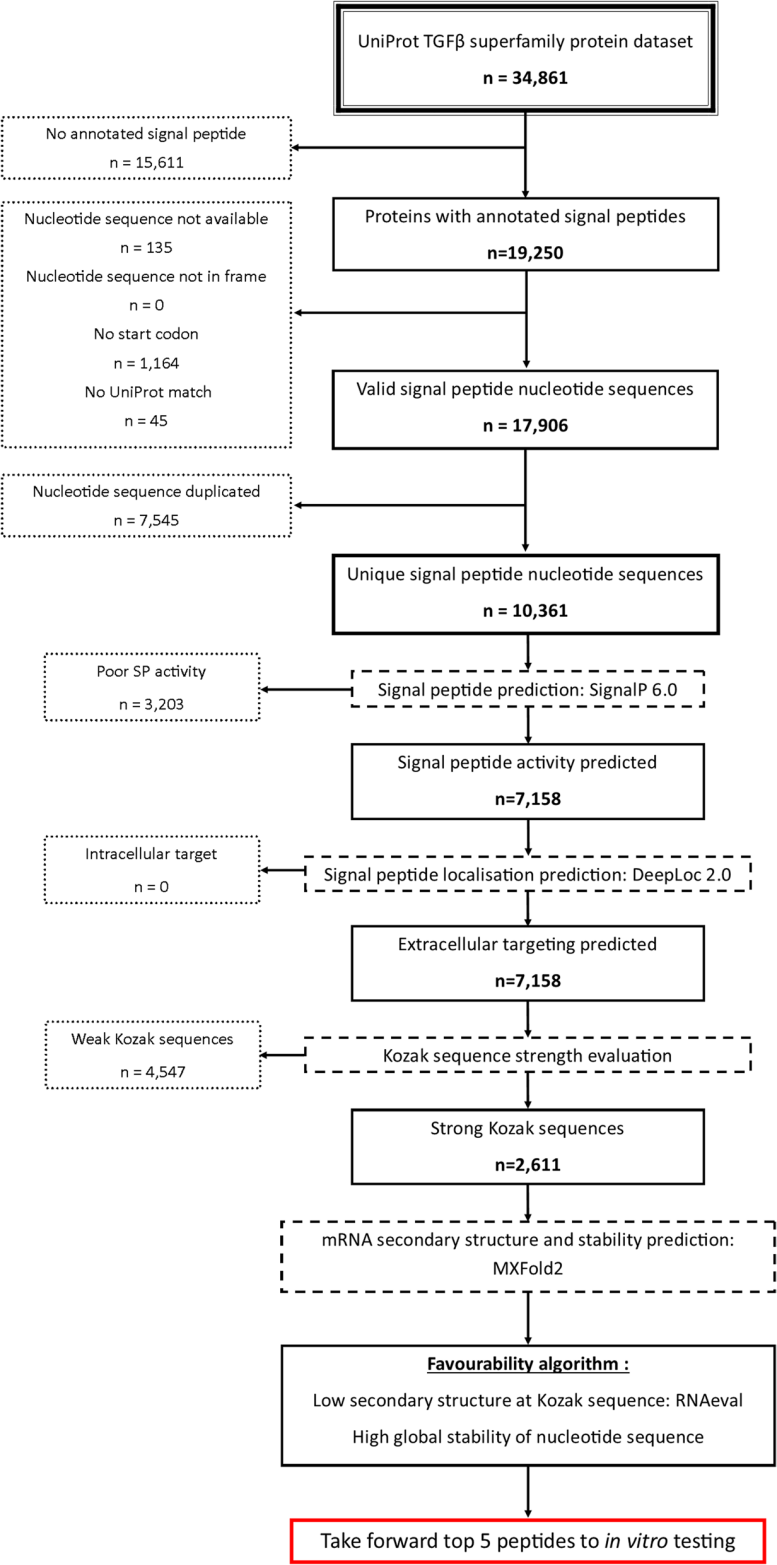
Overview of the filtering process employed in the *in silico* pipeline. Initially ~ 35,000 TGF-β superfamily sequences were retrieved, though only ~ 19,000 were predicted to contain SPs. Of these, ~ 10,000 were found to be valid records and to have unique nucleotide sequences. Existing computational methods indicated ~ 7,000 were predicted to still function and to target the extracellular space when attached to BMP2. ~2,500 were found to have strong Kozak sequences and were taken forward to mRNA structure prediction. Sequences with the least structure at the translational start site but high global stability were thought to be preferable. The top 5 sequences from the pipeline and two manually selected alternatives were taken forward to in vitro work

## Results

### Results from the *in silico* pipeline

The initial TGF-β family dataset retrieved from UniProt contained 34,861 records. 19,250 protein sequences were found to be annotated with a confirmed or likely signal peptide and were taken forward. Nucleotide sequence retrieval and quality control checks (sequence in frame, sequence begins with a start codon, translated nucleotide sequence matches the UniProt protein sequence) were then performed. 1,344 sequences failed one of these criteria and were removed, leaving 17,906 to be taken forward. An SP nucleotide sequence duplicate removal step was then performed, with 7,545 sequences found to be duplicates and removed. 10,361 unique and matched nucleotide/amino acid sequence pairs remained and were used to create the *in silico* fusion protein and predicted mRNA sequence libraries used for further analysis. The *in silico* fusion protein library was subjected to SP activity and localisation prediction. SignalP analysis indicated that 3,203 of the fusion proteins were predicted to have a low chance of secretion and were excluded, leaving 7,158 sequences remaining. DeepLoc analysis indicated all the fusion proteins were predicted to show extracellular secretion, therefore none were excluded. Nucleotide sequences for these fusion proteins were then analysed for weak Kozak sequences. 4,547 sequences were found to have non-optimal Kozak sequences and were excluded. This left a final set of 2,611 sequences for further analysis. See Fig. 1 for an overview of the filtering process.

**Fig. 2:**
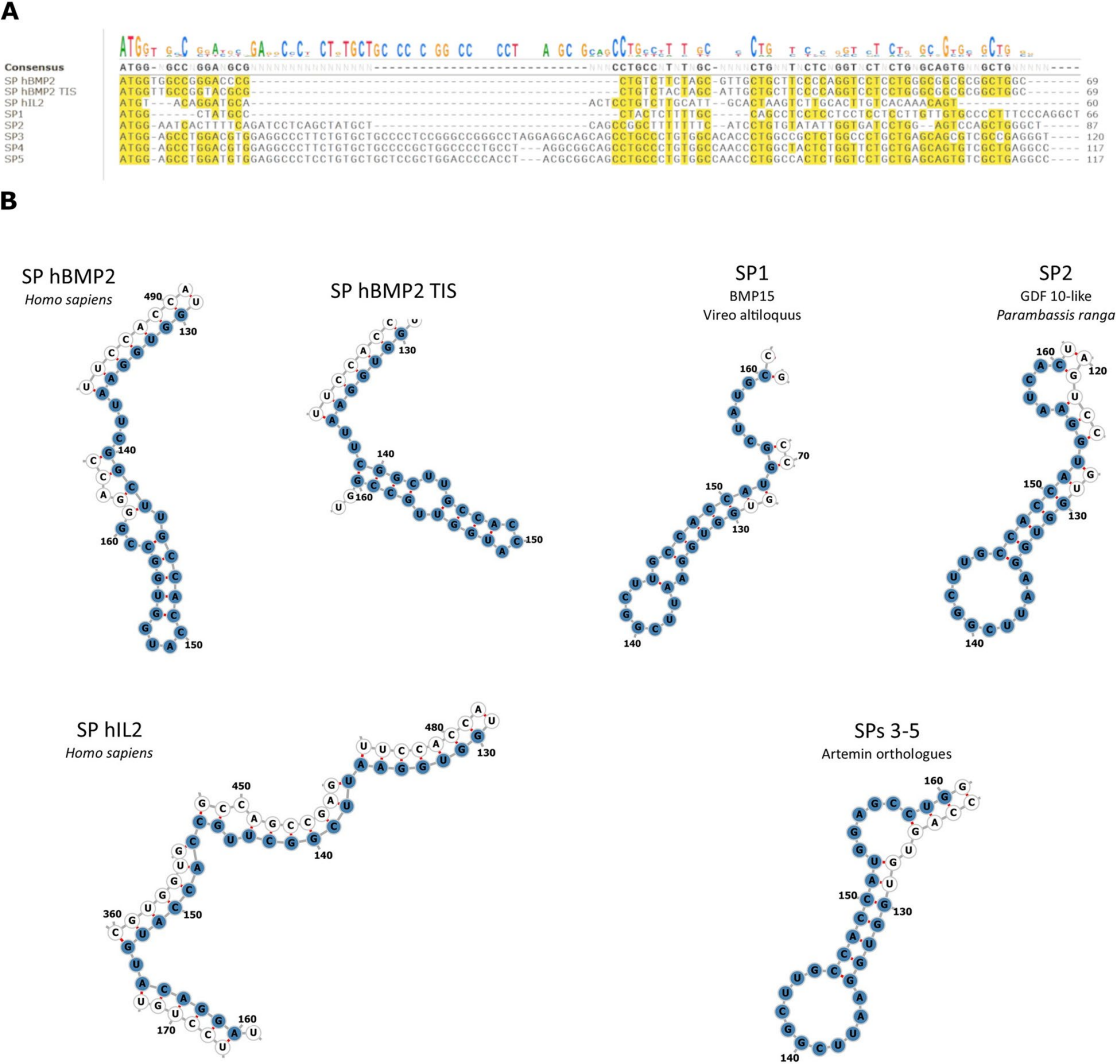
Further nucleotide sequence analysis (**A**) Nucleotide sequence alignment of the final 7 SP sequences. Note the moderate variety with few positions being strongly conserved across the final set. **A** ~ 25 bp insertion can be seen in the SPs from Artemin related genes (SPs 3–5), making them noticeably longer than the other sequences. **B** Predicted mRNA secondary structures at the ribosomal attachment site. The ± 15 bp window is highlighted in blue, with start codon found at positions 151–153

**Fig. 3:**
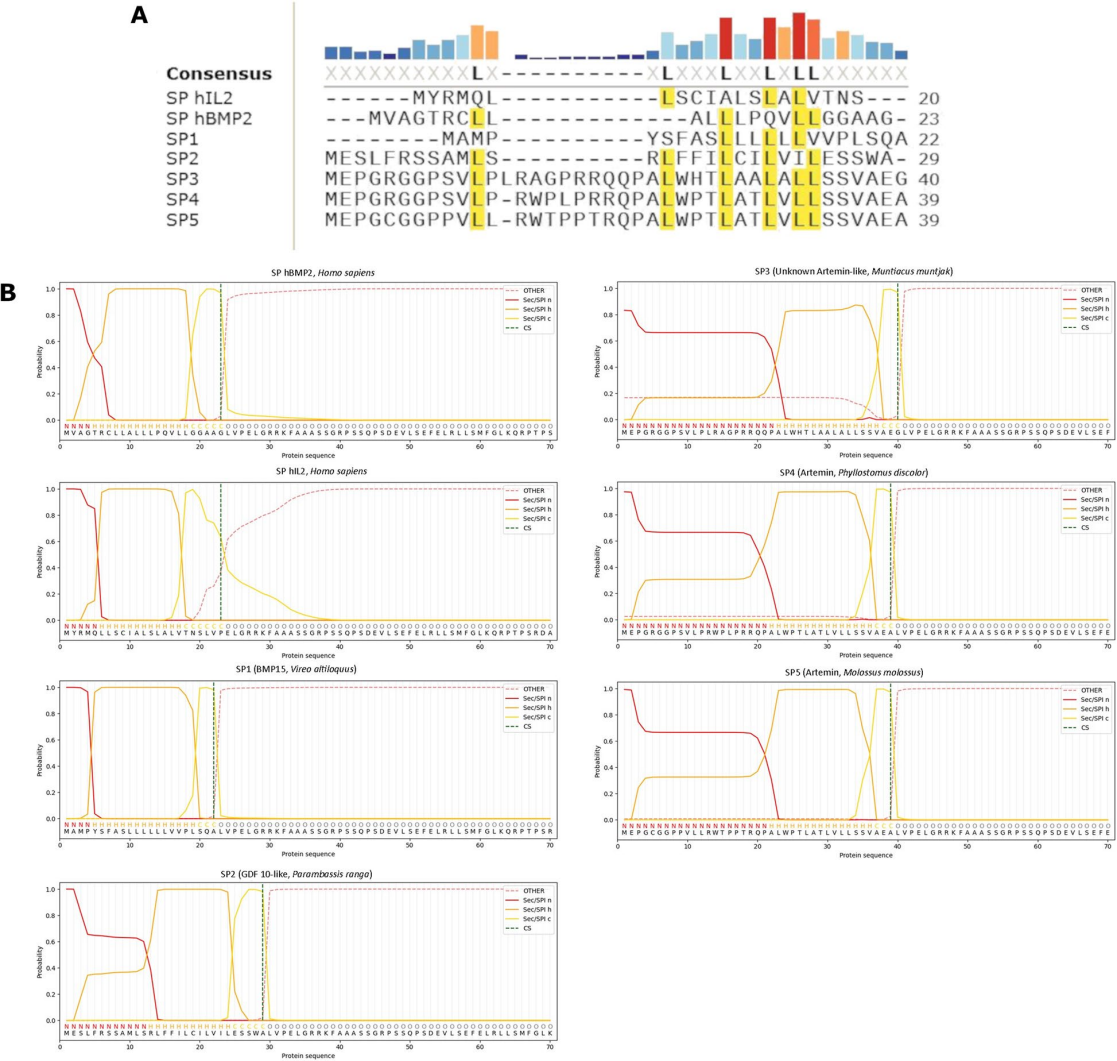
Further protein sequence analysis (**A**) Protein sequence alignment of the final 7 SP sequence. Note the series of conserved leucine residues, the only conserved element across the final panel. Unsurprisingly the three SPs from Artemin related genes (SPs 3–5) were highly similar. **B** Detailed SP region boundary predictions from the SignalP long model. The Artermin related SPs displayed elongated N-regions in comparison to the rest of the set, corresponding to the position of the insertion in the nucleotide sequences (see Fig. 2A). Also note the leucine motif from the alignment is contained within the SP H-region, thought to be important for maintaining hydrophobicity and ensuring the alpha helical conformation required for transmembrane function

The secondary structures of the predicted mRNA sequences for the remaining SP-fusions were predicted with MXFold2. Predicted opening energies varied substantially across the final data set (mean: -343.3 kcal/mol, SD: 106.6), while MFE was much less variable (mean: -451.9 kcal/mol, SD: 11.2). The most promising 5 results from the in silico screen according to their calculated SP score are displayed in Table 1. The sequences were all derived from different vertebrate species. Three sequences were derived from ARTN or closely related unknown protein orthologs, in all cases from mammalian species. The two additional identified proteins were BMP15 and GDF-10 orthologs, from an avian and piscine species respectively. There was modest variation in SP length, ranging from 22 to 44 residues in length (note the native hBMP2 SP is 23 residues long). The MFEs of the 5 sequences showed little variation (mean: -466.7 kcal/mol, SD: 19.6). Opening energies were more variable but covered only a small fraction of the range seen across the whole dataset (mean: -72.6 kcal/mol, SD: 13.6). Sequence alignments revealed that there was limited similarity between sequences from distantly related species and proteins. There was unsurprisingly strong sequence similarity between the 3 artemin orthologs (see Figs. 2A and 3A), with all displaying a ~ 25 bp insertion. All three sequences were predicted to show the same mRNA secondary structure in the ribosomal binding site window (see Fig. 2B). A loose leucin-rich motif was conserved in all sequences (see Fig. 3A).

**Table 1:** Overview of species, protein, sequence data and calculated RNA properties for the top results from the in silico screen

Species	Protein	Abbreviated name	UniProt accession	Nucleotide database & accession	Signal peptide nucleotide sequence (5’ – 3’)	Signal peptide amino acid sequence (*N* – C)	SignalP score	mRNA MFE (kcal/mol)	mRNA opening energy (kcal/mol)	SP score	SP Z-score
*Vireo altiloquus*	BMP 15	SP1	A0A7K5LQ98	EBI ENA: NWT20625.1	ATGGCTATGCCCTACTCTTTTGCCAGCCTCCTCCTCCTCCTCCTTGTTGTGCCCCTTTCCCAGGCT	MAMPYSFASLLLLLLVVPLSQA	0.9997	-438.2	-47	9.32	10.5
*Parambassis ranga*	GDF 10-like	SP2	A0A6P7IG99	NCBI Nucleotide: XM_028407950.1	ATGGAATCACTTTTCAGATCCTCAGCTATGCTCAGCCGGCTTTTTTTCATCCTGTGTATATTGGTGATCCTGGAGTCCAGCTGGGCT	MESLFRSSAMLSRLFFILCILVILESSWA	0.9997	-448.6	-70	6.41	6.6
*Muntiacus muntjak*	Artemin related	SP3	A0A5N3WKB5	EBI ENA: KAB0362191.1	ATGGAGCCTGGACGTGGAGGCCCTTCTGTGCTGCCCCTCCGGGCCGGGCCTAGGAGGCAGCAGCCTGCCCTGTGGCACACCCTGGCCGCTCTGGCCCTGCTGAGCAGCGTCGCCGAGGGT	MEPGRGGPSVLPLRAGPRRQQPALWHTLAALALLSSVAEG	0.5494	-488.7	-82	5.96	6.0
*Phyllostomus discolor*	Artemin	SP4	A0A7E6DRJ2	EBI ENA: KAF6107540.1	ATGGAGCCTGGACGTGGAGGCCCTTCTGTGCTGCCCCGCTGGCCCCTGCCTAGGCGGCAGCCTGCCCTGTGGCCAACCCTGGCTACTCTGGTTCTGCTGAGCAGTGTCGCTGAGGCC	MEPGRGGPSVLPRWPLPRRQPALWPTLATLVLLSSVAEA	0.7878	-480.1	-82	5.85	5.9
*Molossus molossus*	Artemin	SP5	A0A7J8F7B8	EBI ENA: KAF6443102.1	ATGGAGCCTGGATGTGGAGGCCCTCCTGTGCTGCTCCGCTGGACCCCACCTACGCGGCAGCCTGCCCTGTGGCCAACCCTGGCCACTCTGGTCCTGCTGAGCAGTGTCGCTGAGGCC	MEPGCGGPPVLLRWTPPTRQPALWPTLATLVLLSSVAEA	0.6976	-477.9	-82	5.83	5.8

The endogenous hBMP2 SP and the two manually selected sequence, SP hBMP2-TIS and SP hIL2, showed similar MFEs but large opening energies compared to the *in silico* pipeline top 5, with IL2 being particularly high (see Table 2) due to predicted low self-complementarity (see Fig. 2B). Interestingly the hBMP2-TISIGNER sequence, supposedly codon optimised for lowered opening energy, was predicted to have a marginally increased opening energy when compared to the native sequence. This likely results from differences in the methods employed by TISIGNER and MXFold2/RNAEval when predicting structure and assigning bond energy values. All three sequences showed low SP scores, resulting in them acting as useful counterpoints to the top in silico candidates when testing the usefulness of the metric in vitro.

**Table 2:** Overview of species, protein, sequence data and calculated RNA properties for the manually selected sequences

Species	Protein	Abbreviated name	UniProt accession	Nucleotide database & accession	Signal peptide nucleotide sequence (5’ – 3’)	Signal peptide amino acid sequence (*N* – C)	SignalP score	mRNA MFE (kcal/mol)	mRNA opening energy (kcal/mol)	SP score	SP Z-score
*Homo sapiens*	BMP2	SP hBMP2	P12643	NCBI nucleotide: NM_001200.4	ATGGTGGCCGGGACCCGCTGTCTTCTAGCGTTGCTGCTTCCCCAGGTCCTCCTGGGCGGCGCGGCTGGC	MVAGTRCLLALLLPQVLLGGAAG	0.9988	-462.3	-205	2.26	1.0
-	hBMP2 TISIGNER codon optimised	SP hBMP2 TIS	-	-	ATGGTTGCCGGTACGCGCTGTCTACTAGCATTGCTGCTTCCCCAGGTCCTCCTGGGCGGCGCGGCTGGC	MVAGTRCLLALLLPQVLLGGAAG	0.9988	-459.8	-219	2.1	0.8
*Homo sapiens*	IL2	SP hIL2	P60568	NCBI nucleotide: NM_000586.4	ATGTACAGGATGCAACTCCTGTCTTGCATTGCACTAAGTCTTGCACTTGTCACAAACAGT	MYRMQLLSCIALSLALVTNS	0.9994	-435.3	-417	1.04	-0.6

The final seven candidates to be taken forward to in vitro testing were subjected to additional *in silico* analysis. The amino acid sequences were re-submitted to SignalP using the slow  model for an analysis of predicted SP region boundaries. The four sequences that were noticeably longer than the native hBMP2 SP (GFD-10 from *P. ranga* & the artemin orthologs) were predicted to have much longer N-regions than the other sequences (see Fig. 3B). The N-region of hBMP2 is only four residues long, while that of *P. ranga* GDF-10 was 12 residues and the artemin orthologs were 21–22 residues. Additionally, the SP cleavage site for the hIl2 SP was predicted with less confidence than the other sequences and was 3 residues downstream of the true SP/preprotein sequence boundary (see Fig. 3B). The leucine motif identified in the alignment was predicted to be part of the H-region(see Fig. 3B), an established loose feature of mammalian SPs [1].

### *In vitro* validation in two cell lines

**Fig. 4:**
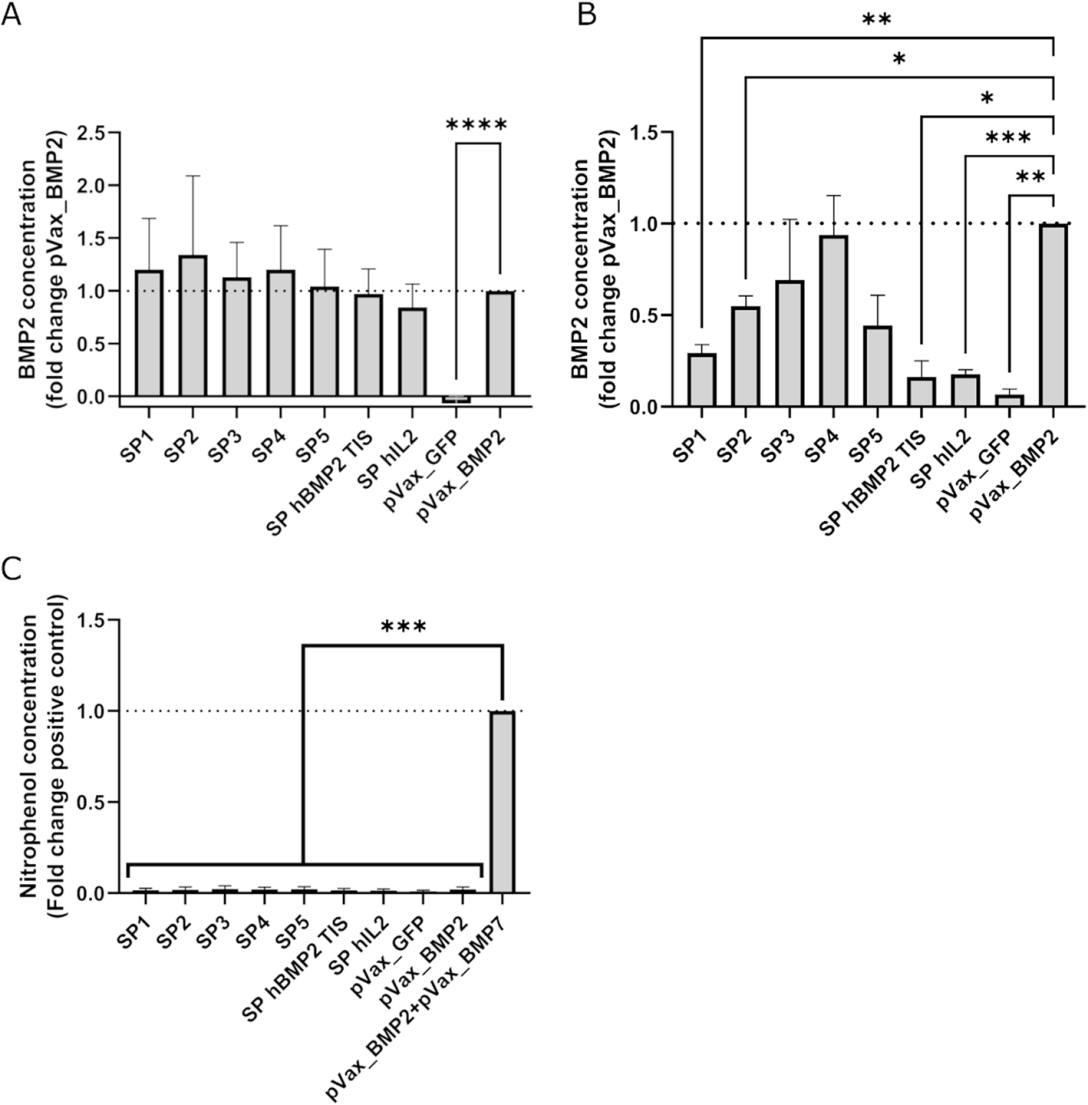
In vitro validation data (A) hBMP2 ELISA data from HEK293T cells. All computationally selected sequences showed non-significant increases in secretion. *N* = 7 ± SD, **** = *p* < 0.0001 (B) hBMP2 ELISA data from C2C12 cells. All computationally selected sequences showed decreased secretion versus the positive control, with several showing significant decreases. Particularly notable were the sequences that showed major decreases in the C2C12 results compared to the HEK293T experiment. These were SP1, SP2, SP hIL2 and SP hBMP2 TIS. *N* = 3 ± SD. * = *p* < 0.05, ** = *p* < 0.01, *** = *p* < 0.001 C) ALP assay data from C2C12 cells. All groups except the positive control showed background levels of ALP activity, indicating the novel SPs were not sufficient to induce osteogenesis in this context. *N* = 3 ± SD, *** = *p* < 0.001

ELISA results from HEK293T conditioned media showed that the signal peptides identified by the *in silico* work and the manually selected sequences of interest did not induce a significant increase in hBMP2 secretion versus the pVax_BMP2 positive control (see Fig. 4A). The *in silico* selected signal peptides all showed small but non-significant increases versus the pVax_BMP2 positive control, while the manually selected SPs showed small and non-significant decreases. Data were highly variable across all groups, with the exception of the pVax_GFP negative control. There appeared to be a minor blanking issue as small negative hBMP2 concentrations were measured for the negative control across all replicates. This was thought to be a minor issue as these values were only a small fraction of that detected in the other samples. It was suspected this was caused by slight differences in blocking behaviour between the fresh complete media used for the standard curve and the conditioned media that made up the samples. C2C12s conditioned media ELISA results showed significant decreases versus the pVax_BMP2 positive control for SPs 1 & 2, SP hBMP2 TISIGNER and SP hIL2, while in silico SPs 3,4 & 5 showed non-significant decreases (see Fig. 4B). After 7 days ALP activity was again at background levels in all groups except the osteogenesis positive control (see Fig. 4C).

**Fig. 5:**
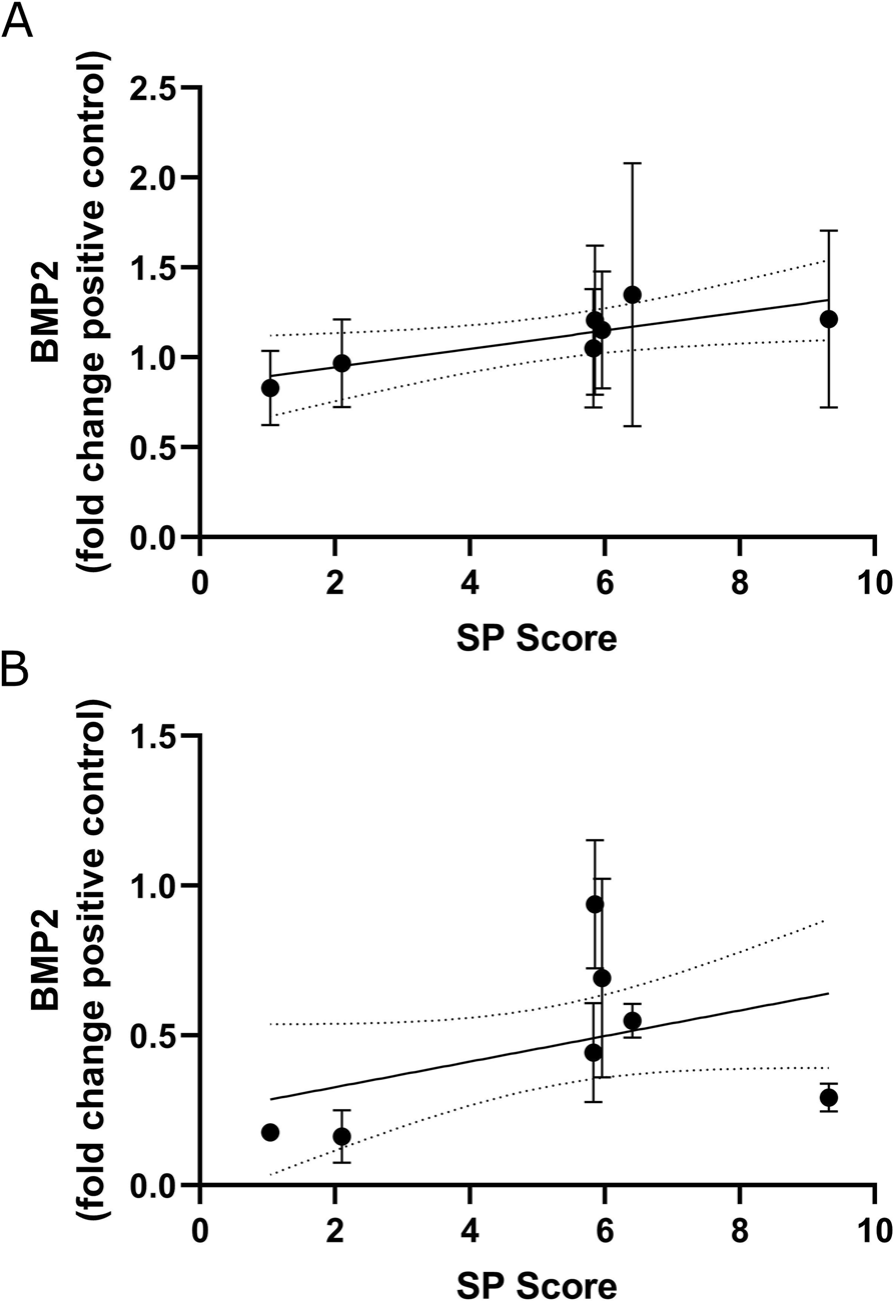
Linear regression analyses of the SP score values and ELISA data (**A**) ELISA data from the HEK239T experiment. Correlation was found to be significant (gradient = 0.0509, R2 = 0.096, F [1, 44] = 4.976, *p* = 0.0305) (**B**) ELISA data from the C2C12 experiment. No significant correlation was found (gradient = 0.0427, R2 = 0.138, F [1, 19] = 3.038, *p* = 0.0975)

Regression analysis of SP scores and ELISA data indicated that there was a significant correlation between SP score and BMP2 secretion in HEK293T (see Fig. 5A; gradient = 0.0509, R^2^ = 0.096, F [1, 44] = 4.976, *p* = 0.0305). No correlation was found between SP score and the heparin supplemented C2C12 ELISA data (see Fig. 5B).

## Discussion

Here we have created an alternative approach to SP prediction, hoping to take advantage of the new generation of computational tools to create quantitative *in silico* predictions of SP effectiveness. We chose to first test the approach with human BMP2, an important osteogenic protein long used in regenerative approach in bone both as a recombinant protein and a gene therapy. Notably BMP2 is known to be a difficult-to-express protein [34, 35], and it was hoped SP engineering might help to alleviate this issue.

While many tools now exist to predict the presence of SPs, they are not capable of making quantitative predictions of how they will perform. To add more utility to the *in silico* segment of the screen than simply filtering out likely negatives (still leaving an overwhelming number of sequences), an additional approach was required. mRNA structure prediction was used to attempt to provide this additional predictive power. Briefly, the signal peptide coding region for SRP SPs contains the start codon and lies only slightly downstream of the ribosomal attachment point. Consequently secondary structure in the region can have a pronounced influence on translation. There is evidence that less stable secondary structure in a ± 15 bp window around the translational start site (which includes the beginning of the SP coding sequence) can improve translation initiation and early elongation [3–5]. Outside the translational start site mRNAs with a higher global stability appear to show improved translation, thought to be due to a combination of improved half-life and the generally higher stability of sequences containing many codons with abundant tRNAs [36, 37]. Consequently, it was though that heterologous SP-BMP2 fusions with mRNAs predicted to display less stable secondary structure at the translation start site and higher global stability might show improved translation and thus more protein export. An mRNA structure and stability based metric, SP score, was devised to assess these two properties simultaneously. It was hypothesised that SP score would correlate with levels of secreted protein.

To test the approach a small subset of the top results from the pipeline and two manually selected sequences were subjected to more detailed *in silico* analysis and tested in vitro. The top five candidates from the pipeline (termed SPs 1–5) were derived from avian, piscine and mammalian species, with SPs drawn from BMP15, GDF10 and three ARTN related genes (see Table 1). The two manually selected sequences were a codon optimised variant of the native hBMP2 SP, and the human IL2 SP. A codon optimised hBMP2 SP allowed examination of the influence of nucleotide sequence changes divorced from changes to the protein sequence. In this case the tool TISIGNER was used to optimise the sequence for reduced opening energy [28], rather than a traditional codon adaption index (cAI) or tRNA adaption index (tAI) based approach. The hIL2 SP was chosen as it is a well-established choice in SP engineering, having been demonstrated to improve secretion for a variety of other proteins [38–42].

In vitro testing in HEK293T showed none of the tested sequences were significantly different from the positive control, and that the data were highly variable (see Fig. 4A). It was suspected that the HEK293T data variability was due to their tendency to form aggregates, leading to increased error during cell seeding which in turn influenced transfection unpredictably [43]. Despite none of the SPs inducing a significant increase in secretion, a correlation between SP score and secreted protein was found (see Fig. 5A), in agreement with the initial hypothesis. This was a promising result and suggested that the mRNA structure predictions had been accurate and had some influence on SP efficacy. Further work with a larger dataset would be required to confirm this fully.

C2C12 ELISA data showed that SPs 1 & 2 and the manually selected sequences showed a significant decreased in secretion versus the positive control, while SPs 3,4 & 5 were not significantly different (see Fig. 4B). There was no correlation between SP score and BMP2 secretion in C2C12 (see Fig. 5B). While the overall results in C2C12 did not agree with the hypothesis, they raised several interesting additional questions. The massive decrease in secretion seen in the two manually selected sequences was particularly interesting. In the case of SP hBMP2 TIS this indicated that the nucleotide sequence was vitally important in this context, as a small number of synonymous mutations almost completely obviated secretion. SP hBMP2 TIS was predicted to show very similar opening energy to the native SP sequence, indicating that either the predictions had been inaccurate or additional factors were at play. Change in codon availability from HEK293T to C2C12 was considered as a possible cause, as the lines are human and murine respectively. A cAI based analysis with ATGme [44] and the murine codon usage table (http://www.kazusa.or.jp/codon/cgi-bin/showcodon.cgi?species=10090), indicating an addition of a single additional low availability codon (< 10%) in the SP hBMP2 TIS versus the native sequence. This was thought to be unlikely to be the sole cause of the massive drop in secretion. Of course, the possibility that the tRNA balance of C2C12 has significantly diverged from the mouse reference data cannot be ignored. An additional possible cause was interaction with antisense oligonucleotides or proteins that are found in C2C12 and not HEK293T. Further investigation would be required to identify these factors.

The SP hIL2 result was also notable as this marks a rare occurrence of the SP not offering improved performance versus a native SP, and indeed reducing it massively [38, 40–42, 39]. It was suspected that this might be related to the predicted change in SP cleavage site for the SP hIL2 fusion. It is well established that multiple SP cleavage sites can be observed using one protein and cell type [45–47], therefore perhaps cleavage at both sites was seen in both lines but the balance between the two sites (or potentially even more than two sites) was shifted. Alternatively, cleavage may have occurred at the correct site but the SP introduced a more subtle alteration to early folding. Why this might occur in C2C12 and apparently not in HEK293T was unknown. Further work using LC-MS/MS would be able to empirically establish the cleavage site/s [45–47].

ALP assay results at day 7 again showed background levels of ALP activity in all groups except the osteogenesis positive control (see Fig. 4C). This indicated that the novel SPs alone were not sufficient to induce an osteogenic effect in C2C12s. This observation was in line with previous data, where BMP2 is known to require either a high concentration or co-expression with other factors such as BMP7 to strongly induce osteogenesis [31, 48–50]. Combined with the ELISA data this was a clear indication that the SPs tested here were not sufficient to improve vector performance. Due to the lack of significant improvement in ELISA and ALP data and the small size of the in vitro data set it was decided to not perform specificity and sensitivity calculations.

There were several clear weaknesses with the approach that were imposed by current limitations in computational methods. While 5’ end mRNA structure likely does play a role in how an SP influences protein secretion, it is certainly not the only factor. Interactions between the SP, SRP machinery and propeptide are known to be important [51–54], however making relevant predictions related to these interactions is difficult with existing computational tools and no established methods exist. While recent advances in multimer tertiary structure prediction by tools such as AlphaFold2 are impressive and have seen some use in modelling SP/translocon interactions [55, 56], modelling of the kinetics of the interactions of multiple multi-subunit complexes is not within current capabilities. If tools able to produce reliable predictions of these interactions are developed, they could perhaps be combined with mRNA structure predictions to improve efficacy. The initial limitation of the sequence panel to the TGF-β superfamily dataset was an attempt to address this problem. It was hoped that SPs from these proteins would be more likely to retain their function in a new but similar context, though it is possible that this was not a reasonable assumption and the dataset was too diverse.

It should also be noted that while RNA structure prediction has seen continuous improvement and a similar burst in progress with machine learning based approaches, predictions are still frequently inaccurate in many contexts [10]. This is thought to be due to biases in the annotated RNA structure data sets used for model training, which are frequently dominated by short ncRNAs and are unable to account for all possible biomacromolecule interactions [57]. Consequently, the mRNA structure predictions used in this approach must be taken with a reasonable degree of scepticism. Advances in high through RNA structure determination techniques promise to address these problems [10, 58, 59], and the ever growing volume of training data from wider contexts promises to allow continued improvement of RNA structure modelling in future. Despite this, the significant correlation observed between SP score and secreted protein in the HEK293T data suggests the predictions were somewhat accurate, though further work would be required to conclusively establish this was not due to chance. Future work could employ a library of synonymous variants to separate the influence of nucleotide sequence from protein structure.

## Conclusions

The mRNA structure-based method for SP effectiveness prediction described here was capable of identifying previously untested SPs capable of functioning comparably to the native sequence in HEK293T. Additionally there was a significant correlation between model predictions and secreted protein in HEK293T, though none of the SPs showed a significant improvement in secretion versus the native SP. The approach was not effective in C2C12 cells with several SPs inducing a significant decrease in secretion. Particularly poor performance from the codon optimised SP hBMP2 TIS indicated the importance of synonymous mutations in the SP and merits further study. These results suggest the mRNA structure prediction approach requires further improvement before it can produce significant improvements in this context, and ideally should be combined with protein sequence based predictions of SP activity in future.

## Data Availability

The datasets used and analysed during the study were publicly available and access links are provided in the main text. Full results from the in silico screen can be provided on request.

## Abbreviations

4NP: 4-Nitrophenol
ALP: Alkaline Phosphetase
BMP2: Bone Morphogenetic Protein 2
MFE: Minimum Free Energy
NPP: Nitrophenol Phosphate
rhBMP2: Recombinant human Bone Morphogenetic Protein 2
Sec/SPI: Sec substrates cleaved by SPase I
SP: Signal Peptide
SRP: Signal Recognition Particle
TISIGNER: Translation initiation coding region designer
